# Optimizing surgical antimicrobial prophylaxis through clinical pharmacist-led audit and feedback: Evidence from a Vietnamese tertiary hospital

**DOI:** 10.1371/journal.pone.0351068

**Published:** 2026-06-26

**Authors:** Dua Thi Nguyen, Son Ngoc Tran, Thu Thi Huyen Nguyen, Thuy Thi Thu Tran, Long Duc Nguyen, Trang Hoang Quynh Nguyen, Hao Trong Nguyen, Shukry Zawahir, Hai Thanh Nguyen, Thuy Thi Thu Nguyen, Van Thi Thuy Pham, Minh Duong Anh Vu, Phuong Thi Xuan Dong, Son Tu Nguyen

**Affiliations:** 1 Saint Paul General Hospital, Hanoi, Vietnam; 2 Faculty of Medicine and Health NHMRC Clinical Trial Center, The University of Sydney, Australia; 3 Faculty of Pharmacology and Clinical Pharmacy, Hanoi University of Pharmacy, Hanoi, Vietnam; University of North Dakota, UNITED STATES OF AMERICA

## Abstract

The inappropriate use of surgical antibiotic prophylaxis (SAP) remains a common issue in Vietnam, posing a significant risk of surgical site infections (SSIs) and other adverse outcomes. This study aimed to evaluate the impact of a pharmacist-led audit–feedback (A&F) intervention, as part of an antimicrobial stewardship (AMS) program, on improving compliance with SAP guidelines. A prospective pre–post interventional study was conducted in the surgical departments of Saint Paul General Hospital. A multidisciplinary team, led primarily by clinical pharmacists, conducted audit–feedback activities on SAP practices from December 2023 to April 2024. The primary outcome was the overall compliance rate with SAP guidelines. Secondary outcomes included compliance with specific components: antibiotic selection, timing of administration, dosing, and duration of use. A total of 162 patients in the pre-intervention group and 147 in the post-intervention group were included. After two audit–feedback phases, overall compliance with SAP guidelines significantly increased from 60.49% to 76.87% (OR: 2.97, 95% CI: 1.71–5.28). Notably, the most significant improvements were observed in the appropriateness of antibiotic selection and duration of use, increasing from 74.69% to 88.44% (OR: 2.57, 95%CI: 1.40–4.89) and 72.84% to 88.44% (OR: 2.82, 95% CI: 1.55–5.36), respectively. Conclusion, the audit–feedback intervention led by clinical pharmacists substantially improved compliance with SAP guidelines across several domains. This study provides pragmatic evidence from a tertiary hospital in Vietnam, demonstrating that audit–feedback is not only feasible but also impactful in resource-limited settings. Importantly, this approach could inform scalable AMS and infection prevention policies in other low-and middle-income countries.

## Introduction

Surgical site infections (SSIs) are among the most common and serious complications following surgical procedures, accounting for a substantial proportion of healthcare-associated infections and contributing to increased postoperative morbidity, prolonged hospital stays, elevated healthcare costs, and even mortality [1,2]. The appropriate use of surgical antimicrobial prophylaxis (SAP)—encompassing appropriate antibiotic selection, optimal timing of administration, and limited duration—has been firmly established as a key preventive measure against SSIs in numerous clinical guidelines [2–4].

Despite the availability of these evidence-based guidelines, studies conducted in both high- and low-to-middle-income countries, including Vietnam, have consistently reported suboptimal compliance to SAP recommendations [5–7]. Common deviations include the use of inappropriate antibiotics, delayed or premature timing of administration, and unnecessarily prolonged postoperative prophylaxis. These non-compliant practices not only diminish the effectiveness of SSI prevention but also contribute to increased antimicrobial resistance (AMR), additional financial burden, and inefficient use of healthcare resources [1].

To address these challenges, audit and feedback (A&F) has been widely endorsed as a core strategy within Antimicrobial Stewardship Programs (ASPs) by numerous international health organizations. Consensus on its importance has been demonstrated by the World Health Organization (WHO) [8], the U.S. Centers for Disease Control and Prevention (CDC) [9], and the UK’s National Institute for Health and Care Excellence (NICE) [10], all of which emphasize A&F as an effective approach to improving antibiotic prescribing practices. Notably, the CDC’s 2019 update to the “Core Elements of Hospital Antibiotic Stewardship Programs” identifies prospective audit with feedback as a key proactive intervention [9].

The effectiveness of A&F interventions in improving SAP compliance has been demonstrated in various healthcare settings. A multicenter study by van Kasteren et al. in the Netherlands showed that structured audit and feedback increased SAP compliance rates from 28% to 68% over two years [11]. Additionally, a systematic review by Davey et al. [12] found that A&F strategies were associated with up to a 20% reduction in inappropriate antibiotic prescribing in hospital settings. In Vietnam, A&F interventions is considered a core strategy in antibiotic stewardship in the national guideline [13]. A recent study has implemented A&F interventions that have proven effective in decreasing inappropriate antimicrobial prescribing and reducing antimicrobial costs in several district hospitals in Vietnam [14].

Clinical pharmacists, equipped with in-depth pharmacological expertise and clinical acumen, play a crucial role in the implementation of A&F interventions. Their ability to engage in interdisciplinary collaboration, monitor antimicrobial prescribing patterns, and provide patient-centered, guideline-concordant recommendations makes them valuable contributors to AMS initiatives, particularly in complex clinical environments such as surgery.

At Saint Paul General Hospital—a major tertiary care hospital in Hanoi—efforts to strengthen AMS infrastructure have been ongoing. Saint Paul General Hospital is a leading institution in surgery in Hanoi. In 2022, the hospital issued guidelines for SAP based on the guidelines of the Vietnamese Ministry of Health [13]. The hospital’s SAP guidelines recommend the administration of beta-lactam antibiotics within 60 minutes before skin incision. Narrow-spectrum antibiotics, e.g., cefazolin, are the preferred choice for most surgeries, with combination regimens used when necessary to broaden the spectrum of activity. The duration of antimicrobial prophylaxis should not exceed 24 hours postoperatively. The implementation became standard practice within one to two years following hospital’s guideline publication. Nonetheless, the matter of compliance with guidelines requires ongoing evaluation and enhancement through pharmacists’ activities, especially A&F. While A&F has been extensively evaluated in high-income settings, evidence from Southeast Asia remains scarce, and no study to date has systematically assessed a clinical pharmacist-led A&F model specifically targeting surgical prophylaxis within a Vietnamese tertiary hospital. Generating local, context-specific evidence is critical to strengthen national AMS initiatives and inform Vietnam’s AMR National Action Plan. This study therefore aimed to evaluate the impact of a structured, clinical pharmacist-led audit–feedback intervention on compliance to surgical prophylaxis guidelines, addressing a key gap in the regional literature.

Specifically, we evaluated changes in antibiotic selection, timing, and duration of prophylaxis pre and post intervention. By generating practical evidence from a real-world tertiary hospital setting, the findings of this study are expected to support the refinement of AMS strategies and contribute to more rational and effective antibiotic use in surgical care. Ultimately, the results may inform national policy-making and enhance patient safety through improved infection prevention and control practices.

## Materials and methods

### Study design and setting

This was a prospective, pre-post interventional study conducted at Saint Paul General Hospital, a 800-bed tertiary care hospital located in Hanoi, Vietnam. Established as one of the leading surgical centers in the capital with nearly 200 beds for surgery departments, the hospital serves as a regional referral institution for surgical cases. The study aimed to evaluate the impact of a clinical pharmacist-led A&F intervention on compliance to the hospital’s SAP guidelines.

### Study population

The study population included patients undergoing elective (scheduled) surgical procedures in the hospital’s surgical departments. Only surgeries that were listed in the hospital’s official SAP guideline and classified as clean or clean-contaminated (as determined by the operating surgeon) were eligible for inclusion. This restriction was based on the scope of the hospital’s SAP guideline, which—at the time of the study—only provided specific recommendations for clean and clean-contaminated surgeries. Therefore, other categories of surgical wounds (e.g., contaminated or dirty) were excluded from the study.

Patients were also excluded if they underwent emergency surgery or had incomplete medical records related to antibiotic use.

### Ethics approval

This study was granted ethics approvals by the Ethics Committee and AMS committee of Saint Paul General Hospital. The guidelines for SAP applied for the study was approved by AMS committee of the hospital. A&F program by pharmacists in the study was also approved by AMS committee of the hospital. Consent, which was waived by the Ethics Committee was not obtained because data were analyzed anonymously. To ensure ethical standards, the authors had no access to information that could identify individual participants during or after data collection.

### Study procedures

**Fig 1:**
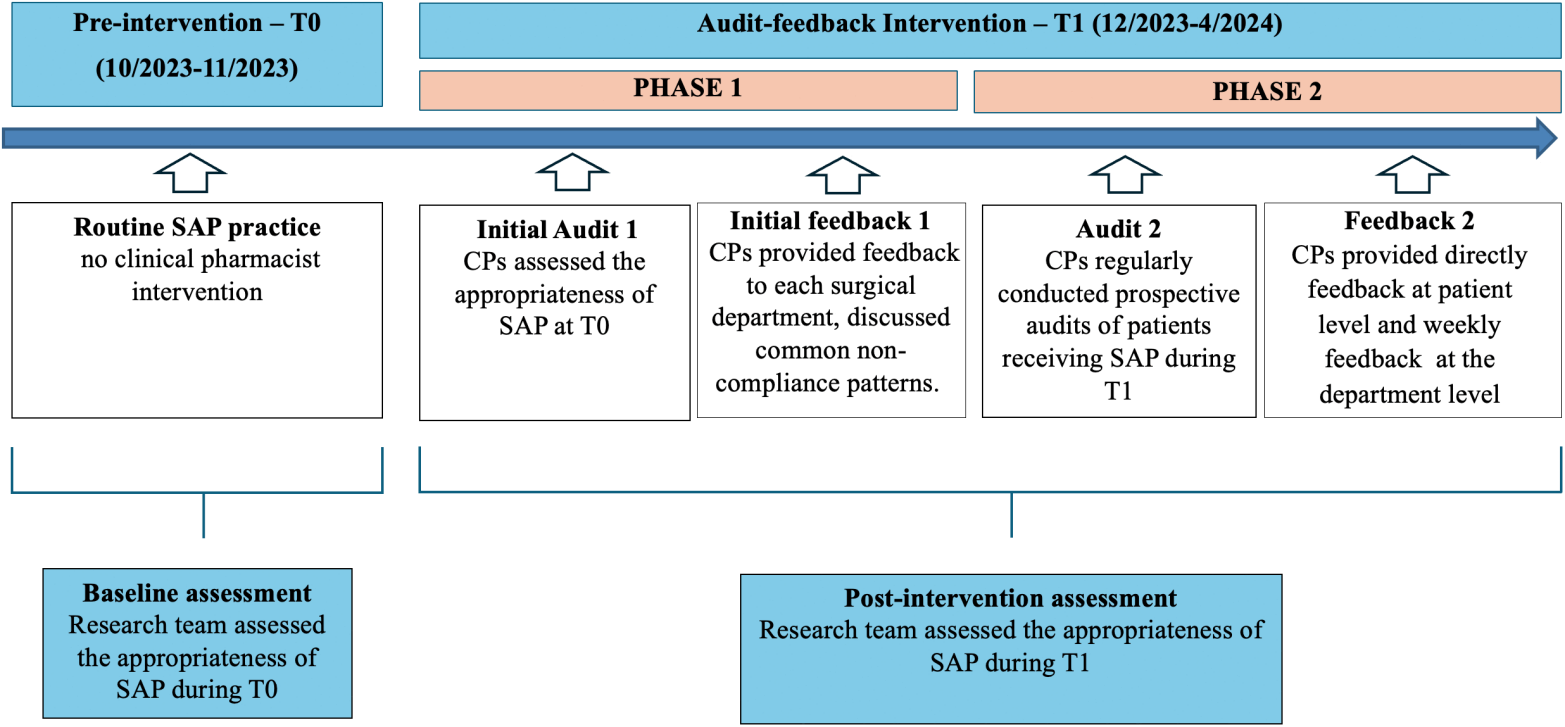
Procedure of the study.

The study procedure is illustrated in Fig 1. Detailed information on the intervention is provided in S1 Table.

### Pre-intervention (initial assessment)

During the pre-intervention phase, prophylactic antibiotics were prescribed independently by surgeons based on the hospital’s internal SAP guideline. This guideline had been developed and approved by the hospital’s AMS committee based on international recommendations and included clear criteria for indications, antibiotic selection, dosing, timing of administration, intraoperative redosing, and duration of use for commonly performed surgerie

From the time the guideline was issued, no intervention, or evaluation regarding its implementation had been conducted. No data was available on actual compliance with the guideline in clinical practice prior to this study.

Therefore, as part of the study design, the research team conducted an initial retrospective assessment of SAP prescribing practices to establish a baseline compliance rate. This assessment served as the reference point (T0) for comparison with post-intervention outcomes. The data collection period was from October 1, 2023, to November 30, 2023 (T0).

### Intervention: Audit and Feedback Program

The A&F intervention was implemented in multiple phases and coordinated by a multidisciplinary stewardship team led by clinical pharmacists. The team comprised clinical pharmacists, infectious disease specialists, and surgical representatives. The data collection period was from December 1, 2023, to April 30, 2024 (T1).

- **Phase 1 – Initial Retrospective Feedback:** Baseline compliance data from the pre-intervention phase were analyzed. Clinical pharmacists presented the results to each surgical department, discussed common non-compliance patterns, and recorded surgeons’ feedback to tailor future interventions.
- **Phase 2 – Prospective Audit and Feedback:** During the post-intervention phase, the stewardship team conducted regularly prospective audits of patients receiving SAP. For each case, antibiotic selection, dose, timing of first dose, and duration of use were assessed. Individualized case-based feedback when needed was provided by clinical pharmacist and weekly summaries of findings were discussed with surgeons at the department level (Fig 1).
- A total of 156 A&F activities (9 weekly department discussion and 147 individual feedback for each cases) were conducted over the 4 months, covering 147 surgical cases.

#### Study outcomes.

The **primary outcome** was the proportion of patients who received fully guideline-compliance of SAP (i.e., appropriate across all five key criteria) before and after the intervention.

**Secondary outcomes** included individual compliance proportion for:

- Antibiotic selection
- Dose administration
- Timing of administration
- Duration of use

These were compared between the pre-intervention (T0) and post-intervention (T1) phases.

- **Sample size calculation**

The sample size was calculated based on the primary outcome: the proportion of patients receiving fully appropriate SAP [15]. Assuming a baseline compliance rate of 50% and an anticipated 30% absolute improvement post-intervention, with a two-sided alpha of 0.05 and 80% power, the required sample size was 130 patients per phase.


$$\begin{array}{c} n=\left(\frac{Pc\;(\text{1}-Pc)}{k}+Pi\;(\text{1}-Pi)\right){\left(\frac{Z_{\text{1}-\alpha }-Z_{\text{1}-\beta }}{Pc-Pi}\right)}^{\text{2}} \end{array}$$

### Data collection and assessment

To minimize assessor bias, data collection was conducted independently from the intervention team during pre-intervention and post-intervention phases. Patient data from electronic medical records and prescriptions were collected by the principal investigator (TS Nguyen) and a postgraduate pharmacy student, both of whom were not involved in the implementation of the audit and feedback intervention.

SAP compliance for each case was assessed using a standardized checklist based on the hospital’s SAP guideline. Additional patient information was collected, including age, sex, comorbidities, surgical procedure, treating department, and infection risk factors.

### Statistical analysis

All data were entered and analyzed using R version 4.2.1. Continuous variables were expressed as mean ± standard deviation (SD) for normally distributed data or median with interquartile range (IQR) for non-normally distributed data. Categorical and ordinal variables were summarized as frequencies and percentages.

To compare variables between the pre- and post-intervention phases:

- Student’s t-test or Mann–Whitney U test was used for continuous variables,
- Chi-square test or Fisher’s exact test was used for categorical variables, as appropriate.

To assess the effect of the intervention on SAP compliance, univariate logistic regression was performed to estimate odds ratios (OR) and 95% confidence intervals (CI). Multivariable logistic regression was then conducted to calculate adjusted ORs, accounting for potential confounding factors such as patient demographics and surgical characteristics. Prior to regression analysis, multicollinearity among variables was assessed using variance inflation factor (VIF), with a VIF greater than 2 considered indicative of multicollinearity. A p-value < 0.05 was considered statistically significant.

## Results

### Patient characteristics

The demographic and clinical characteristics between the two patient groups T0 (N = 162) and T1 (N = 147) showed no statistically significant differences in terms of gender, age, BMI, comorbidities, and number of surgical site infection (SSI) risk factors (p > 0.05). No cases of antibiotic allergy were recorded in either group. Overall, the differences between groups were not statistically significant (Table 1).

**Table 1:** Patient characteristics in the study.

Characteristic	Pre-intervention – T0 (N = 162) n(%)	Post-intervention – T1 (N = 147) n(%)	p-value
**Gender**			
Male	109 (67.28)	90 (61.22)	0.267
Female	53 (32.72)	57 (38.78)	
**Age**			0.497
≤18	61 (37.65)	46 (31.29)	
19–59	64 (39.51)	65 (44.22)	
≥60	37 (22.84)	36 (24.49)	
**BMI**			0.238
<18.5	51 (31.48)	38 (25.85)	
18.5–22.9	58 (35.80)	68 (46.26)	
23.0–24.9	38 (23.46)	26 (17.69)	
≥25	15 (9.26)	15 (10.20)	
**Comorbidities**			0.557
None	151 (93.21)	132 (89.80)	
Diabetes mellitus	6 (3.70)	8 (5.44)	
Other diseases	5 (3.09)	7 (4.76)	
**History of antibiotic allergy**			–
No	162 (100)	147 (100)	

### Surgical characteristics of patients

The surgical characteristics between the two patient groups T0 and T1 also showed no statistically significant differences across all criteria (p > 0.05) (S2 Table). Clean surgeries predominated in both groups (T0: 55.56%, T1: 57.82%), and laparoscopic surgeries accounted for nearly half of the procedures (T0: 40.74%, T1: 45.58%). Blood loss during surgery was below 1500 mL in all patients across both groups. The rate of artificial device implantation was 8.25% in T0 and 4.08% in T1, but the difference was not statistically significant (p = 0.150). The median surgery duration remained stable at 50 minutes in both groups. The majority of procedures lasted less than 60 minutes, while surgeries ≥180 minutes were rare (T0: 3.70%, T1: 2.04%).

### Antibiotic prophylaxis usage

Details of SAP usage during T0 and T1 are summarized in Table 2. The most frequently prescribed prophylactic antibiotic was cefazolin in both phases, accounting for 61.5% at T0 and increasing to 82.7% at T1. The timing of initial antibiotic administration was 30 minutes before surgical incision for all patients, which also represented the shortest preoperative interval observed. The proportion of cases in which SAP was extended beyond 24 hours postoperatively showed a marked reduction after the intervention, declining from 27.16% in T0 to 11.56% in T1.

**Table 2:** Antibiotic prophylaxis usage in the study.

Parameter	Pre-intervention T0 (N = 162)	Post-intervention T1 (N = 147)
**Indication and dose for Adult, n (%)**	**N**_**A0**_ **= 104**	**N**_**A1**_ **= 102**
Cefazolin 2g	64 (61.54)	86 (82.69)
Cefazolin 1g	2 (1.92)	1 (0.96)
Amoxicillin/clavunic acid 2g	19 (18.27)	9 (8.65)
Amoxicillin/clavunic acid 1g	1 (0.96)	–
Ampicillin/sulbactam 3g	1 (0.96)	–
Ampicillin/sulbactam 1.5g	11 (10.58)	1 (0.96)
Cefoperazon/sulbactam 2g	4 (3.85)	–
Cefoperazon/sulbactam 1g	–	2 (1.92)
Cefoperazon/sulbactam 1g + Tinidazole 0.5g	–	2 (1.92)
Ceftriaxon 2g	1 (0.96)	–
Cefamandol 2g	–	1 (0.96)
Cefoperazon/sulbactam 2g + Metronidazole 1g	1 (0.96)	–
**Indication and dose for Children**	**N**_**C0**_ **= 58**	**N**_**C1**_ **= 45**
Cefazolin Frequency, n (%) Dose (mg/kg), median (IQR)	56 (96.55) 31.66 (28.37 - 35.71)	43 (95.56) 30 (26.85 - 33.33)
Amoxicillin/clavunic acid Frequency, n (%) Dose (mg/kg)	–	2 (4.44) (28.57; 43.47)
Ampicilin/sulbactam Frequency, n (%) Dose (mg/kg)	2 (3.45) (17.24; 71.42)	–
**Timing of initial administration**	**N = 162**	**N = 147**
Within 30 minutes prior to surgery	162 (100)	147 (100)
**Duration**	**N = 162**	**N = 147**
Only a single dose prior to surgery	51 (31.48)	47 (31.97)
Extended use within 24 hours post-surgery	67 (41.36)	83 (56.46)
Extended use beyond 24 hours post-surgery	44 (27.16)	17 (11.56)

*Abbreviation:* N_A0_: number of adult patients at phase T0; N_A1_: number of adult patients at phase T1;N_C0_: number of pediatric patients at phase T0; N_C1_: number of pediatric patients at phase T1

### Outcomes

#### Primary outcomes.

Table 3 presents the results of the univariate and multivariate logistic regression analyses for the risk factors associated with full compliance with the SAP guideline (i.e., the primary outcome). A significant improvement was observed after the interventions. In the pre-intervention phase (T0), 60.49% of patients were classified as fully compliant with SAP guidelines, compared with 76.87% in the post-intervention phase (T1) (adjusted OR 2.97, 95% CI: 1.71–5.28, p < 0.001).

**Table 3:** Compliance with SAP Guidelines – Primary Outcomes.

Covariate	Categories	Compliance ratio, n (%)	Crude OR (95% CI)	Adjusted OR (95%CI)	Multivariate P-value
Phase	Pre-intervention phase (T0)	98 (60.49)	1	1	
	Post-intervention (T1)	113 (76.87)	2.17 (1.32-3.56)	2.97 (1.71 - 5.28)	<0.001
Gender	Female	73 (66.36)	1	1	
	Male	138 (69.35)	1.15 (0.697 - 1.89)	1.25 (0.66 - 2.35)	0.4920
Departments	Department group 1*	31 (46.27)	1	1	
	Department group 2*	180 (74.38)	3.37 (1.93 - 5.904)	4.65 (2.42 - 9.20)	<0.001
Age	<=18	79 (73.83)	1	1	–
	19-59	85 (65.89)	0.68 (0.39 - 1.204)	0.77 (0.249 - 2.32)	0.644
	≥60	47 (64.38)	0.64 (0.37 - 1.22)	0.695 (0.215 - 2.22)	0.540
Surgery Risk	0	96 (81.25)	1	1	
	1	167 (60.48)	0.353 (0.194 - 0.642)	0.441 (0.196 - 0.968)	0.0434
	>=2	46 (69.57)	0.527 (0.234 - 1.186)	0.861 (0.268 - 2.80)	0.802
Type of Surgery	Clean	115 (65.71)	1	1	
	Clean-contaminated	96 (71.64)	1.32 (0.81 - 2.148)	1.57 (0.893 - 2.79_	0.122
Surgery approach	Laparoscopic surgery	102 (76.69)	1	1	
	Open surgery	109 (61.93)	0.494 (0.299 - 0.819)	0.617 (0.296 - 1.27)	0.192

Note *: Group 1: Departments with full compliance with the SAP guideline < 50% at T0, Group 2: Departments with full compliance with the SAP guideline ≥ 50% at T0.

#### Secondary outcomes.

The compliance rate with SAP guidelines in T1 showed a significant improvement compared to T0 across multiple criteria (Table 4). Specifically, the “Antibiotic selection” criterion increased from 74.69% to 88.44% (p = 0.002, OR = 2.57, 95% CI: 1.40–4.89), and “Duration of use” increased from 72.84% to 88.44% (p < 0.001, OR = 2.82, 95% CI: 1.55–5.36). The criteria “Timing of administration” and “Additional dosing” maintained 100% compliance, demonstrating stable compliance to these aspects.

**Table 4:** Compliance with SAP Guidelines – Secondary Outcomes.

Criteria	Pre-intervention T0 (N = 162) n(%)	Post-intervention T1 (N = 147) n(%)	p-value	OR (95% CI)
Antibiotic selection	121 (74.69)	130 (88.44)	0.002	2.57 (1.40 - 4.89)
Dosage	138 (85.19)	133 (90.48%)	0.157	1.64 (0.82 - 3.40)
Timing of administration	162 (100)	147 (100)	–	–
Duration of use	118 (72.84)	130 (88.44)	<0.001	2.82 (1.55 - 5.36)

### Compliance with SAP Guidelines by department

**Fig 2:**
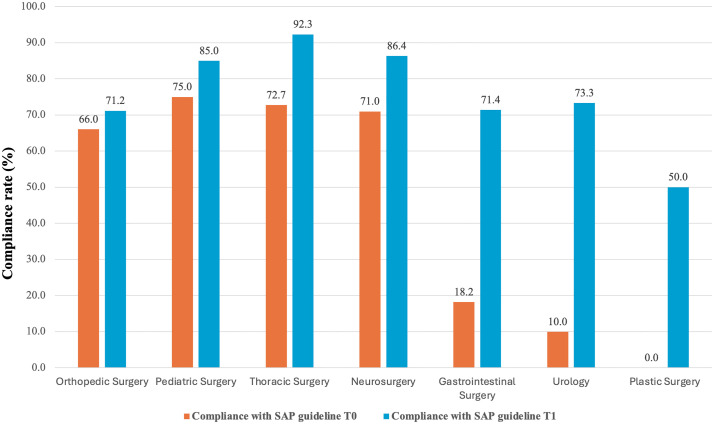
Compliance with SAP guideline by department at pre and post-intervention.

Fig 2 shows compliance rates with SAP guidelines increased in most departments after the intervention, with particularly notable improvements in some specialties. Specifically, the Gastrointestinal Surgery department increased from 18.18% (2/11) to 71.43% (15/21), with p = 0.008, and the Urology department increased from 10.00% (1/10) to 73.33% (11/15), with p = 0.004. These represent statistically significant improvements. Additionally, the Plastic Surgery department increased from 0.00% (0/6) to 50.00% (2/4), although this change was not statistically significant (p = 0.133). These improvements are particularly important in gastrointestinal and urologic surgeries, where the risk of surgical site infections is highest, and where inappropriate prolonged prophylaxis has been a persistent challenge.

## Discussion

This study demonstrates that a clinical pharmacist-led audit–feedback program can rapidly improve compliance to SAP guidelines in a Vietnamese tertiary hospital (nearly 200 beds for surgery departments). The primary outcomes in this study were process-of-care indicators reflecting adherence to SAP guideline recommendations, including appropriate antibiotic selection, dose, timing, and duration. These measures are commonly used in antimicrobial stewardship research because they directly assess the quality of perioperative antimicrobial practice and are sensitive to changes introduced by stewardship interventions. Improved adherence to these indicators may ultimately contribute to more appropriate antimicrobial use and better patient outcomes. Within four months, overall compliance rose from 62% to 82%, with notable gains in antibiotic selection and duration. To our knowledge, this is the first pharmacist-led SAP stewardship intervention in Vietnam, and among the few reported from Southeast Asia. The findings not only carry intrinsic value for enhancing local clinical practice but also contribute to the global evidence base supporting the audit–feedback strategy—recognized as a core component of antimicrobial stewardship (AMS) programs recommended by the World Health Organization (WHO), particularly suitable for low- and middle-income countries such as Vietnam [8].

### Discussion on the audit–feedback strategy

Audit–feedback is considered a key strategy within AMS [8,13]. This strategy has been shown to be effective not only in antibiotic stewardship but also in other medication groups for modifying healthcare workers’ practices [16]. In the context of antibiotic stewardship, audit feedback has been extensively implemented in various studies as a primary intervention, especially in low- and middle-income countries, to influence prescribers [17]. Additionally, the Ministry of Health of Vietnam has identified audit feedback as a crucial component of the national antimicrobial stewardship guidelines [13]. The hospital pharmacists provided direct feedback to clinical departments and prescribers in both phases, aiming to modify behaviors and practices to align with SAP guidelines. In developing countries like Vietnam, this strategy is cost-effective, requiring minimal infrastructure investment compared to other interventions [8]. Moreover, it serves as an educational tool, particularly for junior physicians, enhancing their accountability and evidence-based prescribing practices. The impact of this strategy can be directly observed through increased rates of appropriate antibiotic use, improved patient outcomes, reduced hospital costs, and enhanced multidisciplinary collaboration within the AMS program [8]. This strategy has also been applied and shown effective in other antibiotic stewardship studies in Vietnam [14].

In this present study, the audit and feedback intervention was deliberately structured in multiple phases to address different objectives and maximize the sustainability of practice change. **Phase 1 (Initial Retrospective Feedback)** aimed to build awareness and foster consensus by presenting baseline compliance data to surgeons, highlighting common non-compliance patterns, and collecting feedback to tailor subsequent strategies. **Phase 2 (Prospective Audit and Feedback)** focused on reinforcing behavior change and maintaining compliance over time, through regular department-level discussions of audit findings and individualized case-based feedback when necessary. This multi-phase approach allowed the stewardship team to sequentially (i) raise awareness of current practice gaps, (ii) sustain continuous improvement through regular reinforcement, and (iii) adapt feedback messages to evolving clinical practice, thereby enhancing both the short-term impact and long-term sustainability of the intervention [8,17].

### Baseline comparability of study populations across pre and post intervention

Given the pre–post intervention study design, ensuring baseline comparability of the study populations across the two phases is essential. The analysis showed no statistically significant differences in demographic, clinical, or surgical characteristics between the T0 and T1 groups (p > 0.05). Importantly, the groups were also similar regarding surgical site infection (SSI) risk factors, such as surgery type and implant use. This comparability eliminates confounding from baseline characteristics, thereby strengthening the internal validity and attribution of the improvement in SAP compliance to the audit–feedback intervention.

### Magnitude of improvement in SAP guideline compliance

The improvement observed in this study aligns with and extends findings from previous research on audit–feedback interventions for surgical prophylaxis [12,17]. In terms of magnitude of effect, our intervention produced a 19% absolute increase in overall compliance (from 60.49% to 76.87%) within a four-month period. This improvement is comparable to other studies in Vietnam, the post-intervention compliance rate in this study was significantly higher than in Huu Nghi Hospital (24.3%) [18], Thong Nhat Hospital (59.3%) [19], 108 Military Hospital (65.93%) [20]. While cross-hospital comparisons must consider institutional differences, it is noteworthy that these facilities are high-tier public hospitals —suggesting that this intervention may be especially impactful when adapted to provincial hospital contexts.

When compared to the landmark multicenter study by van Kasteren et al. (2003) in the Netherlands, which reported an increase in SAP compliance from 28% to 68% over 2 years, our 16.4% increase within 4 months is notable [11]. A 2017 Cochrane review by Davey et al. also found that audit–feedback programs reduced inappropriate prescribing by 20% on average [12]. Thus, the improvement observed here aligns with global averages and demonstrates the replicability of audit–feedback results in the Vietnamese public hospital setting—even with limited resources.

When examining department-level effects, the largest improvements were seen in gastrointestinal and urologic surgery, where compliance rose from 18.2% to 71.4% and 10% to 73.3%, respectively. These specialties are associated with clean-contaminated procedures and higher baseline risk for surgical site infections, underscoring the importance of targeted stewardship interventions in these departments. Such focused improvements demonstrate that audit–feedback can deliver disproportionate benefits in high-risk surgical areas, complementing international evidence that stewardship interventions are most impactful when applied to vulnerable patient populations.

### Shifts in antibiotic selection and duration

Another important finding was the positive shift in antibiotic selection and post-operative duration. Prescribers increasingly favored narrow-spectrum agents, especially cefazolin monotherapy, while significantly reducing the use of broad-spectrum antibiotics like third-generation cephalosporins and betalactam/betalactam inhibitors. Overuse of broad-spectrum agents in clean and clean-contaminated surgeries has been documented in prior Vietnamese studies [7,21]. This results in a higher risk of developing AMR, causing adverse events in patients, and incurring unnecessary treatment costs [22]. Meanwhile, the use of cefazolin is also considered a cost-saving measure for patients, as this antibiotic is less expensive than the aforementioned broad-spectrum options.

Post-operative antibiotic duration also improved, with compliance to recommended duration rising from 72.84% to 88.44%. Inappropriate extension of antibiotics until hospital discharge is common in Vietnam’s public hospitals [7,18,23], whereas current guidelines recommend discontinuation within 24 hours for these surgeries [13]. This improvement reflects increased clinician trust in and adoption of evidence-based hospital guidelines.

More importantly, these changes were most evident in gastrointestinal and urologic surgery departments—both associated with clean-contaminated procedures and endogenous microbial risk—highlighting areas where targeted stewardship can have significant impact.

### Sustainability and the role of clinical pharmacists

In AMS programs, pharmacists play a vital role in shaping prescribers’ behavior through various interventions. These include education, training, guideline development, direct communication, and audit-feedback [8,17]. At Saint Paul Hospital, the SAP guideline was issued in 2022. However, merely issuing a guideline seems insufficient to ensure good compliance with prophylactic antibiotic use. This has been observed in several hospitals in Vietnam where antimicrobial stewardship programs with appropriate strategies have not yet been implemented [18,24]. This underlines the necessity of periodic audit–feedback interventions to maintain real-world compliance, beyond the passive dissemination of policy documents.

In response, clinical pharmacists initiated the AMS team’s involvement and proposed hospital-wide audit–feedback in all surgical departments. For sustainable impact and broader implementation, deeper understanding of prescriber behaviors is needed—particularly regarding combination therapy, preference for broad-spectrum cephalosporins, and rationale for prolonged prophylaxis. Such insights would inform more refined interventions aimed at optimizing SAP in terms of both clinical efficacy and cost-efficiency.

Sustaining the long-term effectiveness of antimicrobial prophylaxis interventions likely requires multifaceted strategies, including ongoing education, audit and feedback, and system-based approaches. In this context, the integration of digital technologies, such as electronic health record (EHR)-based prompts, may help reinforce and maintain adherence to prophylaxis guidelines over time [8,12]. However, the implementation of such system-level solutions remains limited by infrastructure and resource constraints, and may not yet be widely feasible across healthcare institutions. Therefore, the AMS committee and clinical pharmacy services play a crucial role in sustaining appropriate antimicrobial use through audit and feedback activities. The achieved improvement stemmed from coordinated efforts—systematic intervention guided by hospital leadership, and regular clinical pharmacy activities at the ward level. Everyday, clinical pharmacists and the study team reviewed and analyzed SAP use by department and delivered direct feedback to physicians. This routine not only served as a reminder but also facilitated bidirectional communication, enabling prescribers to better understand non-compliance issues and receive immediate support for correction. Physician engagement was a decisive factor in the success and smooth execution of the audit–feedback process.

### Limitations

The study has several limitations. First, post-discharge clinical outcomes were not assessed, making it impossible to link improved compliance with reduced SSI rates. Future studies should incorporate follow-up of SSI incidence to strengthen the causal connection between compliance and patient safety. Second, given the pre–post design, there is potential for unmeasured confounding factors. External influences such as staff turnover, seasonal variation in surgical case mix, or national AMR awareness campaigns could have contributed to changes in prescribing practices. Although baseline characteristics were comparable across phases, causality should be interpreted cautiously. Third, the short follow-up period (four months) limits insights into long-term sustainability. Improvements achieved through audit–feedback may wane without ongoing reinforcement, and periodic monitoring will likely be required to maintain compliance.

## Conclusions

This study demonstrates that a clinical pharmacist-led audit–feedback intervention substantially improved compliance to surgical antimicrobial prophylaxis (SAP) guidelines in a Vietnamese tertiary hospital. As the first pharmacist-led SAP stewardship study in Vietnam, these findings provide locally relevant evidence that audit–feedback is both feasible and effective in a resource-limited setting. The intervention leveraged pharmacists’ expertise and multidisciplinary collaboration to deliver rapid, measurable improvements in prescribing practices.

These results have important policy implications, suggesting that audit–feedback could be integrated into national antimicrobial stewardship and infection prevention programs across low – and middle -income countries. Sustained implementation, coupled with evaluation of clinical outcomes such as surgical site infections and cost-effectiveness, will be essential to consolidate and extend these gains.

## Supporting information

S1 Table TIDieR Checklist for the intervention. (DOCX)

S2 Table Surgical characteristics of patients in the study. (DOCX)

## Abbreviations

A&F: Audit – Feedback
AMS: Antimicrobial Stewardship
AMR: Antimicrobial Resistance
HAI: Healthcare-Associated Infection
IQR: Interquartile Range
OR: Odd Ratio
SAP: Surgical Antibiotic Prophylaxis
SSI: Surgical Site Infections
WHO: World Health Organization
