# Supplementary material for: Optimizing surgical antimicrobial prophylaxis through clinical pharmacist-led audit and feedback: Evidence from a Vietnamese tertiary hospital
Source: PLoS One. 2026 Jun 26;21(6):e0351068. doi: 10.1371/journal.pone.0351068 (PMC13308818; doi:10.1371/journal.pone.0351068)
Supplement: S1 Table — (DOCX) [file pone.0351068.s001.docx]

**Supplementary 1 Table. TIDieR Checklist for the Intervention**

| **Item (TIDieR domain)** | **Description of intervention in this study** |
| --- | --- |
| **1. Brief name** | Clinical pharmacist–led audit and feedback (A&F) program for surgical antimicrobial prophylaxis (SAP). |
| **2. Why (rationale)** | Inappropriate SAP practices (especially prolonged duration and broad-spectrum antibiotic use) were identified as key contributors to antimicrobial resistance. Audit and feedback, led by clinical pharmacists, aimed to improve adherence to hospital SAP guidelines. |
| **3. What (materials)** | Standardized **audit forms/checklists** based on hospital SAP criteria; written **audit reports**; |
| **4. What (procedures)** | Clinical pharmacists prospectively reviewed surgical cases against SAP criteria. Noncompliance was flagged and discussed. Feedback provided in two formats:  **(a) case-based** discussions within 24 h  **(b) aggregate feedback** during weekly departmental meetings. |
| **5. Who provided** | Three trained **clinical pharmacists**, each assigned to specific surgical departments, supervised by AMS teams and supported by the hospital antimicrobial stewardship committee. |
| **6. How (delivery mode)** | Feedback delivered **face-to-face** (bedside, morning rounds, postoperative debriefings) and during **group meetings**. Written reports circulated electronically to department heads. |
| **7. Where (location)** | Surgical wards, and meeting rooms at Saint Paul Hospital, Hanoi, Vietnam. |
| **8. When and how much** | Intervention implemented continuously for **4 months** (Dec 2023–Apr 2024). Case-based feedback provided **daily**; aggregate feedback **weekly per department.** |
| **9. Tailoring (adaptation)** | Feedback customized by department: e.g., focus on antibiotic selection and duration in gastrointestinal /urologic surgery, on timing/redosing in anesthesia |
| **10. Modifications** | No major protocol changes; intensity of bedside feedback increased in departments with persistently low compliance. |
| **11. Fidelity (intervention adherence)** | Fidelity monitored via logs of number of cases audited, and acceptance rate of pharmacist recommendations. Periodic review by the AMS team ensured consistency. |
| **12. How well (planned/actual)** | Planned fidelity monitoring was achieved: ≥90% of audited cases received timely feedback, and departmental reports were delivered every week as scheduled. |
