# Supplementary material for: Optimizing surgical antimicrobial prophylaxis through clinical pharmacist-led audit and feedback: Evidence from a Vietnamese tertiary hospital
Source: PLoS One. 2026 Jun 26;21(6):e0351068. doi: 10.1371/journal.pone.0351068 (PMC13308818; doi:10.1371/journal.pone.0351068)
Supplement: S2 Table — (DOCX) [file pone.0351068.s002.docx]

**S2 Table. **Surgical Characteristics of Patients in the study****

| **haracteristic** | **Pre- intervention - T0**  **(N=162)** | **Post- intervention - T1**  **(N=147)** | **p-value** |
| --- | --- | --- | --- |
| **Type of surgery** |  |  |  |
| Clean | 90 (55.56%) | 85 (57.82%) | 0.104 |
| Clean-contaminated | 72 (44.44%) | 62 (42.18%) |  |
| **Surgical approach** |  |  |  |
| Open surgery | 96 (59.26%) | 80 (54.42%) | 0.391 |
| Laparoscopic surgery | 66 (40.74%) | 67 (45.58%) |  |
| **Number of SSI risk factors** |  |  | 0.280 |
| 0 | 52 (32.09%) | 39 (26.53%) |  |
| 1 | 90 (55.56%) | 79 (53.74%) |  |
| 2 | 16 (9.87%) | 25 (17.01%) |  |
| ≥3 | 4 (2.47%) | 4 (2.72%) |  |
| **Intraoperative blood loss** |  |  |  |
| ≥1500 mL | 0 (0.00%) | 0 (0.00%) | – |
| <1500 mL | 162 (100.00%) | 147 (100.00%) |  |
| **Implantation of artificial device** |  |  |  |
| Yes | 13 (8.25%) | 6 (4.08%) | 0.150 |
| **Surgical department** |  |  | 0.058 |
| Orthopedic surgery | 53 (37.72%) | 52 (35.37%) |  |
| Pediatric surgery | 40 (24.69%) | 20 (13.61%) |  |
| Thoracic surgery | 11 (6.79%) | 13 (8.84%) |  |
| Neurosurgery | 31 (19.14%) | 22 (14.97%) |  |
| Gastrointestinal surgery | 11 (6.79%) | 21 (14.28%) |  |
| Urologic surgery | 10 (6.17%) | 15 (10.20%) |  |
| Plastic surgery | 6 (3.70%) | 4 (2.72%) |  |
| **Duration of surgery (minutes)** |  |  | 0.221 |
| Median (IQR) | 50 (30–270) | 50 (30–200) |  |
| <60 minutes | 87 (53.7%) | 77 (52.38%) |  |
| 60–119 minutes | 55 (33.95%) | 61 (41.50%) |  |
| 120–179 minutes | 14 (8.64%) | 6 (4.08%) |  |
| ≥180 minutes | 6 (3.7%) | 3 (2.04%) |  |
