# Supplementary material for: Optimizing surgical antimicrobial prophylaxis through clinical pharmacist-led audit and feedback: Evidence from a Vietnamese tertiary hospital
Source: PLoS One. 2026 Jun 26;21(6):e0351068. doi: 10.1371/journal.pone.0351068 (PMC13308818; doi:10.1371/journal.pone.0351068)
Supplement: Attachment — Submitted filename: Response to editor and reviewers.docx [file pone.0351068.s003.docx]

**Response letter to comments by editor and reviewers**

**Journal: PLOS One**

**Manuscript title: Optimizing Surgical Antimicrobial Prophylaxis through Clinical Pharmacist-Led Audit and Feedback: Evidence from a Vietnamese Tertiary Hospital**

**EDITOR’S COMMENTS**

**Editor’s** **Comment 1. Please ensure that your manuscript meets PLOS ONE's style requirements, including those for file naming**

*Response:*

Thank you for this reminder.

We have carefully reviewed the manuscript and all associated files to ensure full compliance with PLOS ONE’s style requirements, including file naming conventions.

**Editor’s** **Comment 2**. **We note that you have included the phrase “from unpublished pilot data” in your manuscript. Unfortunately, this does not meet our data sharing requirements. PLOS does not permit references to inaccessible data. We require that authors provide all relevant data within the paper, Supporting Information files, or in an acceptable, public repository. Please add a citation to support this phrase or upload the data that corresponds with these findings to a stable repository (such as Figshare or Dryad) and provide and URLs, DOIs, or accession numbers that may be used to access these data. Or, if the data are not a core part of the research being presented in your study, we ask that you remove the phrase that refers to these data.**

*Response:*

Thank you for pointing this out.

We acknowledge that the phrase “from unpublished pilot data” does not comply with PLOS data availability requirements. As these pilot data are not essential to the core findings of the present study, we have removed this phrase from the manuscript to ensure full compliance with the journal’s policies.

The revised manuscript has been updated accordingly (page 08, line 181).

We appreciate the editor’s guidance in maintaining adherence to data transparency standards.

**Editor’s Comment 3. Please note that there are currently two tables labeled as "Table 3" on pages 13 and 15. Please renumber your tables and update the corresponding citations in the text to ensure they follow a sequential order.**

*Response:*

Thank you for pointing out this inconsistency.

We have carefully reviewed the manuscript and corrected the duplicate table numbering. The tables have now been renumbered to ensure a consistent and sequential order throughout the manuscript. In addition, all corresponding in-text citations have been updated accordingly to reflect these changes.

The revisions have been made on page 14 (line 252 and 257) of the manuscript.

We appreciate the editor’s attention to detail.

**Editor’s Comment 4. Please include captions for all your Supporting Information files at the end of your manuscript, and update any in-text citations to match accordingly. Please see our Supporting Information guidelines for more information:** [**http://journals.plos.org/plosone/s/supporting-information**](http://journals.plos.org/plosone/s/supporting-information)**.**

*Response:*

Thank you for this helpful suggestion.

We have revised the manuscript to include captions for all Supporting Information files at the end of the document, in accordance with PLOS Supporting Information guidelines. Each file is now clearly labeled and accompanied by a descriptive caption.

In addition, all in-text citations referring to Supporting Information have been reviewed and updated to ensure consistency with the revised file labels. For the readers' convenience, we have also inserted two figures (Fig 1 and 2) from the appendix beneath the relevant paragraphs.

The corresponding changes have been made on page 6 (line 134-137), page 15 (268-270) of the manuscript.

**Editor’s Comment 5. We note that there is identifying data in the Supporting Information file <SAP Data.xlsx>. Please remove or anonymize all personal information (Ages more specific than whole numbers, patient ID numbers, BMI), ensure that the data shared are in accordance with participant consent, and re-upload a fully anonymized data set. Please note that spreadsheet columns with personal information must be removed and not hidden as all hidden columns will appear in the published file.**

*Response:*

Thank you for highlighting this important issue regarding data anonymization.

In response, we have carefully reviewed the Supporting Information file (SAP Data) and removed all potentially identifying information, including patient ID numbers and any indirect identifiers (e.g., removed detailed age, weight, height data where applicable). New variable named “age group” was applied instead of “age”. All variables have been assessed to ensure that the dataset is fully anonymized and compliant with participant consent and PLOS data sharing policies.

We have also ensured that any columns containing personal or potentially identifiable information have been completely removed (rather than hidden), in accordance with the journal’s requirements.

A revised, fully anonymized version of the dataset has now been uploaded.

We appreciate the editor’s guidance in ensuring compliance with data protection standards.

**REVIEWER 1 COMMENTS**

**Reviewer 1 Comment 1. As the authors mentioned, post-discharge clinical outcomes were not assessed and the study was likely not powered to look at those outcomes. This did not bother me since the appropriate application of surgical antimicrobial processes have been shown to reduce the incidence of SSI.**

*Response:*

We thank the reviewer for this thoughtful comment and for recognizing the scope and intent of our study.

We appreciate the reviewer’s acknowledgment that optimizing antimicrobial processes remains a meaningful and evidence-based target for improving surgical outcomes.

**Reviewer 1 Comment 2. I would characterize the antimicrobial prophylaxis metrics in this study to be principally process of care measures (you either did it correctly or did not).**

*Response:*

We thank the reviewer for this insightful observation.

We agree that the antimicrobial prophylaxis metrics evaluated in our study can be considered process-of-care measures. In our manuscript, these metrics were described as “compliance with guidelines,” reflecting whether clinical practice adhered to established recommendations for surgical antimicrobial prophylaxis.

To improve conceptual clarity and align with the reviewer’s comment, we have revised the manuscript to explicitly state that these “compliance with guidelines” metrics represent process-of-care quality indicators. This clarification has been incorporated in the Discussion sections (page 15, lines 274-280).

We appreciate the reviewer’s suggestion, which has helped strengthen the conceptual framing of our study.

**Reviewer 1 Comment 3. Although the use of clinical pharmacists to perform audit and feedback clearly worked to improve care related to antimicrobial prophylaxis, I question whether it will be sustainable over the long term versus making structural changes such as standing orders, EHR prompts, or others to prevent backsliding related to surgical practices.**

*Response:*

We thank the reviewer for this important comment regarding the sustainability of audit-and-feedback interventions.

We agree that structural interventions, such as standardized order sets or electronic health record (EHR)-based prompts, may offer more sustainable mechanisms to support appropriate antimicrobial prophylaxis in the long term. However, in the context of our study setting in Vietnam, the implementation of such system-level solutions remains limited by infrastructure and resource constraints, and may not yet be widely feasible across healthcare institutions.

Importantly, our findings should be interpreted within the context of a healthcare system that is currently undergoing a transition toward more guideline-based clinical practice. Following a prolonged period during which concerns about infection prevention and control influenced more conservative or variable antimicrobial use, there is now increasing emphasis on aligning practice with evidence-based recommendations.

In this setting, audit and feedback delivered by clinical pharmacists represents a pragmatic and context-appropriate strategy to facilitate behavior change. Rather than enforcing compliance through rigid system constraints, this approach supports prescribers through professional dialogue, clinical reasoning, and persuasive communication—mechanisms that may be particularly relevant in promoting acceptance and gradual cultural change in prescribing practices.

We have added a discussion to reflect these considerations and to highlight the need for future integration of structural interventions, as system capacity evolves (Discussion, page 20, lines 385-392).

We appreciate the reviewer’s valuable perspective on the long-term sustainability of such interventions.

**REVIEWER 2 COMMENTS**

**Reviewer 2 Comment 1. Please consider consistently using SAP as an abbreviation in the manuscript. As surgical antibiotic prophylaxis has already been described as SAP in the abstract (Line 19), I would recommend using the abbreviation instead of the full term on Line 34 (..."improved compliance with surgical antibiotic prophylaxis..."), Line 81 and Line 118.**

*Response:* Thank you for pointing out these abbreviation issues.

We have carefully reviewed the manuscript and used SAP as an abbreviation throughout the manuscript (line 37, 85 and 128)

**Reviewer 2 Comment 2. Introduction: Line 47 - change appropriateness to appropriate for clarity**

*Response:* Thank you for pointing out these typographical and formatting issues.

We have carefully reviewed the manuscript and corrected all typographical errors and inconsistencies throughout the text

**Reviewer 2 Comment 3. On Line 82, there is a reference to the Vietnam Ministry of Health guidelines. I found accessing the guidelines difficult. Would it be possible to briefly mention some of the key points in the guideline (e.g. common antibiotic choice or recommended duration of use) in the manuscript for readers to use a reference when reviewing results of the study?**

*Response:*

We thank the reviewer for this helpful suggestion.

We agree that providing additional context on the Vietnam Ministry of Health guidelines would improve the interpretability of our findings for international readers. In response, we have added a brief summary of key recommendations (including common antibiotic choices and recommended duration of prophylaxis) in the main text (page 04, lines 86–90).

We appreciate the reviewer’s suggestion, which has helped enhance the clarity and accessibility of our manuscript.

**Reviewer 2 Comment 4. Line 213: should "phas" be phases?**

*Response:* Thank you for pointing out these typographical and formatting issues.

We have carefully reviewed the manuscript and corrected all typographical errors and inconsistencies throughout the text.

**Reviewer 2 Comment 5. Line 280: Change "perspective" to prospective**

*Response:* Thank you for pointing out these typographical and formatting issues.

We have carefully reviewed the manuscript and corrected all typographical errors and inconsistencies throughout the text.
