# Supplementary material for: Optimizing surgical antimicrobial prophylaxis through clinical pharmacist-led audit and feedback: Evidence from a Vietnamese tertiary hospital
Source: PLoS One. 2026 Jun 26;21(6):e0351068. doi: 10.1371/journal.pone.0351068 (PMC13308818; doi:10.1371/journal.pone.0351068)
Supplement: Peer review history

# Peer review: Decision Letter 0

**DOI:** 10.1371/journal.pone.0351068.r001

**Authors:** Tze Shien Lo

13 Apr 2026

PONE-D-25-68518

Optimizing Surgical Antimicrobial Prophylaxis through Clinical Pharmacist-Led Audit and Feedback: Evidence from a Vietnamese Tertiary Hospital

PLOS One

Dear Dr. Nguyen,

Thank you for submitting your manuscript to PLOS ONE. After careful consideration, we feel that it has merit but does not fully meet PLOS ONE’s publication criteria as it currently stands. Therefore, we invite you to submit a revised version of the manuscript that addresses the points raised during the review process.

Please submit your revised manuscript by May 28 2026 11:59PM. If you will need more time than this to complete your revisions, please reply to this message or contact the journal office at plosone@plos.org. When you're ready to submit your revision, log on to https://www.editorialmanager.com/pone/ and select the ’submissions Needing Revision' folder to locate your manuscript file.

Please include the following items when submitting your revised manuscript:

- A letter that responds to each point raised by the academic editor and reviewer(s). You should upload this letter as a separate file labeled 'Response to Reviewers'.
- A marked-up copy of your manuscript that highlights changes made to the original version. You should upload this as a separate file labeled 'Revised Manuscript with Track Changes'.
- An unmarked version of your revised paper without tracked changes. You should upload this as a separate file labeled 'Manuscript'.

If you would like to make changes to your financial disclosure, please include your updated statement in your cover letter. Guidelines for resubmitting your figure files are available below the reviewer comments at the end of this letter.

If applicable, we recommend that you deposit your laboratory protocols in protocols.io to enhance the reproducibility of your results. Protocols.io assigns your protocol its own identifier (DOI) so that it can be cited independently in the future. For instructions see: https://journals.plos.org/plosone/s/submission-guidelines#loc-laboratory-protocols. Additionally, PLOS ONE offers an option for publishing peer-reviewed Lab Protocol articles, which describe protocols hosted on protocols.io. Read more information on sharing protocols at https://plos.org/protocols?utm_medium=editorial-email&utm_source=authorletters&utm_campaign=protocols.

As the corresponding author, your ORCID iD is verified in the submission system and will appear in the published article. PLOS supports the use of ORCID, and we encourage all coauthors to register for an ORCID iD and use it as well. Please encourage your coauthors to verify their ORCID iD within the submission system before final acceptance, as unverified ORCID iDs will not appear in the published article. *Only*   the individual author can complete the verification step; PLOS staff *cannot*   verify ORCID iDs on behalf of authors.

We look forward to receiving your revised manuscript.

Kind regards,

Tze Shien Lo, MD

Academic Editor

PLOS One

Journal Requirements:

When submitting your revision, we need you to address these additional requirements.

1. Please ensure that your manuscript meets PLOS ONE’s style requirements, including those for file naming. The PLOS ONE style templates can be found at

https://journals.plos.org/plosone/s/file?id=wjVg/PLOSOne_formatting_sample_main_body.pdf and https://journals.plos.org/plosone/s/file?id=ba62/PLOSOne_formatting_sample_title_authors_affiliations.pdf

2. We note that you have included the phrase “from unpublished pilot data” in your manuscript. Unfortunately, this does not meet our data sharing requirements. PLOS does not permit references to inaccessible data. We require that authors provide all relevant data within the paper, Supporting Information files, or in an acceptable, public repository. Please add a citation to support this phrase or upload the data that corresponds with these findings to a stable repository (such as Figshare or Dryad) and provide and URLs, DOIs, or accession numbers that may be used to access these data. Or, if the data are not a core part of the research being presented in your study, we ask that you remove the phrase that refers to these data.

3. Please note that there are currently two tables labeled as "Table 3" on pages 13 and 15. Please renumber your tables and update the corresponding citations in the text to ensure they follow a sequential order.

4. Please include captions for all your Supporting Information files at the end of your manuscript, and update any in-text citations to match accordingly. Please see our Supporting Information guidelines for more information: http://journals.plos.org/plosone/s/supporting-information.

5. We note that there is identifying data in the Supporting Information file <SAP Data.xlsx>. Due to the inclusion of these potentially identifying data, we have removed this file from your file inventory. Prior to sharing human research participant data, authors should consult with an ethics committee to ensure data are shared in accordance with participant consent and all applicable local laws.

Data sharing should never compromise participant privacy. It is therefore not appropriate to publicly share personally identifiable data on human research participants. The following are examples of data that should not be shared:

-Name, initials, physical address

-Ages more specific than whole numbers

-Internet protocol (IP) address

-Specific dates (birth dates, death dates, examination dates, etc.)

-Contact information such as phone number or email address

-Location data

-ID numbers that seem specific (long numbers, include initials, titled “Hospital ID”) rather than random (small numbers in numerical order)

Data that are not directly identifying may also be inappropriate to share, as in combination they can become identifying. For example, data collected from a small group of participants, vulnerable populations, or private groups should not be shared if they involve indirect identifiers (such as sex, ethnicity, location, etc.) that may risk the identification of study participants.

Additional guidance on preparing raw data for publication can be found in our Data Policy (https://journals.plos.org/plosone/s/data-availability#loc-human-research-participant-data-and-other-sensitive-data) and in the following article: http://www.bmj.com/content/340/bmj.c181.long.

Please remove or anonymize all personal information (Ages more specific than whole numbers, patient ID numbers, BMI), ensure that the data shared are in accordance with participant consent, and re-upload a fully anonymized data set. Please note that spreadsheet columns with personal information must be removed and not hidden as all hidden columns will appear in the published file.

If the reviewer comments include a recommendation to cite specific previously published works, please review and evaluate these publications to determine whether they are relevant and should be cited. There is no requirement to cite these works unless the editor has indicated otherwise.

Please review your reference list to ensure that it is complete and correct. If you have cited papers that have been retracted, please include the rationale for doing so in the manuscript text, or remove these references and replace them with relevant current references. Any changes to the reference list should be mentioned in the rebuttal letter that accompanies your revised manuscript. If you need to cite a retracted article, indicate the article’s retracted status in the References list and also include a citation and full reference for the retraction notice.

Additional Editor Comments:

We have now received all of the peer reviewers' reports for your manuscript. The reviewers have found the work interesting and of value; however, they have raised some minor points that need to be addressed before the manuscript can be considered for publication.

Please carefully read the comments and recommendations written by the reviewers and make the corrections as recommended or respond to the recommendations with a rebuttal.

[Note: HTML markup is below. Please do not edit.]

Reviewers' comments:

Reviewer’s Responses to Questions

**Comments to the Author**

1. Is the manuscript technically sound, and do the data support the conclusions?

The manuscript must describe a technically sound piece of scientific research with data that supports the conclusions. Experiments must have been conducted rigorously, with appropriate controls, replication, and sample sizes. The conclusions must be drawn appropriately based on the data presented.

Reviewer #1: Yes

Reviewer #2: Yes

**********

2. Has the statistical analysis been performed appropriately and rigorously?

Reviewer #1: Yes

Reviewer #2: Yes

**********

3. Have the authors made all data underlying the findings in their manuscript fully available?

The PLOS Data policy requires authors to make all data underlying the findings described in their manuscript fully available without restriction, with rare exception (please refer to the Data Availability Statement in the manuscript PDF file). The data should be provided as part of the manuscript or its supporting information, or deposited to a public repository. For example, in addition to summary statistics, the data points behind means, medians and variance measures should be available. If there are restrictions on publicly sharing data—e.g. participant privacy or use of data from a third party—those must be specified.

Reviewer #1: Yes

Reviewer #2: Yes

**********

4. Is the manuscript presented in an intelligible fashion and written in standard English?

PLOS ONE does not copyedit accepted manuscripts, so the language in submitted articles must be clear, correct, and unambiguous. Any typographical or grammatical errors should be corrected at revision, so please note any specific errors here.

Reviewer #1: Yes

Reviewer #2: Yes

**********

5. Review Comments to the Author

Please use the space provided to explain your answers to the questions above. You may also include additional comments for the author, including concerns about dual publication, research ethics, or publication ethics. (Please upload your review as an attachment if it exceeds 20,000 characters)

Reviewer #1: Surgical site infections (SSI) remain common among surgical inpatients occurring in up to 3% of all operations. SSIs result in increased costs of care, reoperations, ICU care and patient death. One of the important strategies for prevention of SSI is appropriate antimicrobial prophylaxis. Despite multiple guideline recommendations the inappropriate use of broad-spectrum antimicrobials remains common and prolonged duration of antimicrobials continues for many operations.

In this study, Nguyen and colleagues conducted a study of pharmacist-driven audit and feedback intervention to change antimicrobial prescribing practices for surgery at a tertiary care hospital in Hanoi. Using a pre-, post-intervention study design, the authors demonstrated a significant improvement in a composite measure of surgical antimicrobial prophylaxis. The improvement was greatest for two types of surgery – gastrointestinal and urologic surgery (both of which had the greatest opportunity for improvement). The two process measures that improved the most were antimicrobial selection for surgery and the duration of antimicrobial use after surgery. Patients undergoing elective operations or those where medical records were incomplete were not included in the study. Only operations categorized as clean or clean-contaminated were included in the study. While all of the metrics captured were process of care measures, the authors captured information on patient characteristics and types of operations to calculate adjusted odds ratios for compliance with antimicrobial prophylaxis guidelines. The authors did not capture post-discharge outcomes measures such as surgical site infection rates or other clinical outcomes.

The study has a number of strengths:

• The focus of the study was on the processes of care related to antimicrobial surgical prophylaxis. The power calculation to look at study enrollment was appropriate and the study was sufficiently powered to evaluate the compliance with the antibiotic guidelines.

• The authors used audit and feedback as the intervention in the study which is an appropriate tool to use to improve antibiotic stewardship metrics. Audit and feedback has been used in many interventions to improve quality of care.

• The details of the interventions by the pharmacy team was appropriate and staged to start with general education followed by personal assessment of practices at the level of the surgeon.

I had few weaknesses:

• As the authors mentioned, post-discharge clinical outcomes were not assessed and the study was likely not powered to look at those outcomes. This did not bother me since the appropriate application of surgical antimicrobial processes have been shown to reduce the incidence of SSI.

• I would characterize the antimicrobial prophylaxis metrics in this study to be principally process of care measures (you either did it correctly or did not).

• Although the use of clinical pharmacists to perform audit and feedback clearly worked to improve care related to antimicrobial prophylaxis, I question whether it will be sustainable over the long term versus making structural changes such as standing orders, EHR prompts, or others to prevent backsliding related to surgical practices.

Reviewer #2: Dear authors,

Thank you for providing the opportunity to review your work. AMR continues to be a global issue, thus any approach to enhance prescribing and administration of antibiotics should be commended. I have very few minor suggestions for consideration (as most of my queries were progressively addressed throughout the manuscript):

1. Please consider consistently using SAP as an abbreviation in the manuscript. As surgical antibiotic prophylaxis has already been described as SAP in the abstract (Line 19), I would recommend using the abbreviation instead of the full term on Line 34 (..."improved compliance with surgical antibiotic prophylaxis..."), Line 81 and Line 118.

2. Introduction: Line 47 - change appropriateness to appropriate for clarity

3. On Line 82, there is a reference to the Vietnam Ministry of Health guidelines. I found accessing the guidelines difficult. Would it be possible to briefly mention some of the key points in the guideline (e.g. common antibiotic choice or recommended duration of use) in the manuscript for readers to use a reference when reviewing results of the study?

4. Line 213: should "phas" be phases?

5. Line 280: Change "perspective" to prospective

**********

6. PLOS authors have the option to publish the peer review history of their article (what does this mean?). If published, this will include your full peer review and any attached files.

If you choose “no”, your identity will remain anonymous but your review may still be made public.

**Do you want your identity to be public for this peer review?**   For information about this choice, including consent withdrawal, please see our Privacy Policy.

Reviewer #1: **Yes:**  Dale Bratzler

Reviewer #2: No

**********

[NOTE: If reviewer comments were submitted as an attachment file, they will be attached to this email and accessible via the submission site. Please log into your account, locate the manuscript record, and check for the action link "View Attachments". If this link does not appear, there are no attachment files.]

To ensure your figures meet our technical requirements, please review our figure guidelines: https://journals.plos.org/plosone/s/figures

You may also use PLOS’s free figure tool, NAAS, to help you prepare publication quality figures: https://journals.plos.org/plosone/s/figures#loc-tools-for-figure-preparation.

NAAS will assess whether your figures meet our technical requirements by comparing each figure against our figure specifications.

# Author comment: Author response to Decision Letter 1

**DOI:** 10.1371/journal.pone.0351068.r002

10 May 2026

EDITOR’S COMMENTS

Editor’s Comment 1. Please ensure that your manuscript meets PLOS ONE’s style requirements, including those for file naming

Response:

Thank you for this reminder.

We have carefully reviewed the manuscript and all associated files to ensure full compliance with PLOS ONE’s style requirements, including file naming conventions.

Editor’s Comment 2. We note that you have included the phrase “from unpublished pilot data” in your manuscript. Unfortunately, this does not meet our data sharing requirements. PLOS does not permit references to inaccessible data. We require that authors provide all relevant data within the paper, Supporting Information files, or in an acceptable, public repository. Please add a citation to support this phrase or upload the data that corresponds with these findings to a stable repository (such as Figshare or Dryad) and provide and URLs, DOIs, or accession numbers that may be used to access these data. Or, if the data are not a core part of the research being presented in your study, we ask that you remove the phrase that refers to these data.

Response:

Thank you for pointing this out.

We acknowledge that the phrase “from unpublished pilot data” does not comply with PLOS data availability requirements. As these pilot data are not essential to the core findings of the present study, we have removed this phrase from the manuscript to ensure full compliance with the journal’s policies.

The revised manuscript has been updated accordingly (page 08, line 181).

We appreciate the editor’s guidance in maintaining adherence to data transparency standards.

Editor’s Comment 3. Please note that there are currently two tables labeled as "Table 3" on pages 13 and 15. Please renumber your tables and update the corresponding citations in the text to ensure they follow a sequential order.

Response:

Thank you for pointing out this inconsistency.

We have carefully reviewed the manuscript and corrected the duplicate table numbering. The tables have now been renumbered to ensure a consistent and sequential order throughout the manuscript. In addition, all corresponding in-text citations have been updated accordingly to reflect these changes.

The revisions have been made on page 14 (line 252 and 257) of the manuscript.

We appreciate the editor’s attention to detail.

Editor’s Comment 4. Please include captions for all your Supporting Information files at the end of your manuscript, and update any in-text citations to match accordingly. Please see our Supporting Information guidelines for more information: http://journals.plos.org/plosone/s/supporting-information.

Response:

Thank you for this helpful suggestion.

We have revised the manuscript to include captions for all Supporting Information files at the end of the document, in accordance with PLOS Supporting Information guidelines. Each file is now clearly labeled and accompanied by a descriptive caption.

In addition, all in-text citations referring to Supporting Information have been reviewed and updated to ensure consistency with the revised file labels. For the readers' convenience, we have also inserted two figures (Fig 1 and 2) from the appendix beneath the relevant paragraphs.

The corresponding changes have been made on page 6 (line 134-137), page 15 (268-270) of the manuscript.

Editor’s Comment 5. We note that there is identifying data in the Supporting Information file <SAP Data.xlsx>. Please remove or anonymize all personal information (Ages more specific than whole numbers, patient ID numbers, BMI), ensure that the data shared are in accordance with participant consent, and re-upload a fully anonymized data set. Please note that spreadsheet columns with personal information must be removed and not hidden as all hidden columns will appear in the published file.

Response:

Thank you for highlighting this important issue regarding data anonymization.

In response, we have carefully reviewed the Supporting Information file (SAP Data) and removed all potentially identifying information, including patient ID numbers and any indirect identifiers (e.g., removed detailed age, weight, height data where applicable). New variable named “age group” was applied instead of “age”. All variables have been assessed to ensure that the dataset is fully anonymized and compliant with participant consent and PLOS data sharing policies.

We have also ensured that any columns containing personal or potentially identifiable information have been completely removed (rather than hidden), in accordance with the journal’s requirements.

A revised, fully anonymized version of the dataset has now been uploaded.

We appreciate the editor’s guidance in ensuring compliance with data protection standards.

REVIEWER 1 COMMENTS

Reviewer 1 Comment 1. As the authors mentioned, post-discharge clinical outcomes were not assessed and the study was likely not powered to look at those outcomes. This did not bother me since the appropriate application of surgical antimicrobial processes have been shown to reduce the incidence of SSI.

Response:

We thank the reviewer for this thoughtful comment and for recognizing the scope and intent of our study.

We appreciate the reviewer’s acknowledgment that optimizing antimicrobial processes remains a meaningful and evidence-based target for improving surgical outcomes.

Reviewer 1 Comment 2. I would characterize the antimicrobial prophylaxis metrics in this study to be principally process of care measures (you either did it correctly or did not).

Response:

We thank the reviewer for this insightful observation.

We agree that the antimicrobial prophylaxis metrics evaluated in our study can be considered process-of-care measures. In our manuscript, these metrics were described as “compliance with guidelines,” reflecting whether clinical practice adhered to established recommendations for surgical antimicrobial prophylaxis.

To improve conceptual clarity and align with the reviewer’s comment, we have revised the manuscript to explicitly state that these “compliance with guidelines” metrics represent process-of-care quality indicators. This clarification has been incorporated in the Discussion sections (page 15, lines 274-280).

We appreciate the reviewer’s suggestion, which has helped strengthen the conceptual framing of our study.

Reviewer 1 Comment 3. Although the use of clinical pharmacists to perform audit and feedback clearly worked to improve care related to antimicrobial prophylaxis, I question whether it will be sustainable over the long term versus making structural changes such as standing orders, EHR prompts, or others to prevent backsliding related to surgical practices.

Response:

We thank the reviewer for this important comment regarding the sustainability of audit-and-feedback interventions.

We agree that structural interventions, such as standardized order sets or electronic health record (EHR)-based prompts, may offer more sustainable mechanisms to support appropriate antimicrobial prophylaxis in the long term. However, in the context of our study setting in Vietnam, the implementation of such system-level solutions remains limited by infrastructure and resource constraints, and may not yet be widely feasible across healthcare institutions.

Importantly, our findings should be interpreted within the context of a healthcare system that is currently undergoing a transition toward more guideline-based clinical practice. Following a prolonged period during which concerns about infection prevention and control influenced more conservative or variable antimicrobial use, there is now increasing emphasis on aligning practice with evidence-based recommendations.

In this setting, audit and feedback delivered by clinical pharmacists represents a pragmatic and context-appropriate strategy to facilitate behavior change. Rather than enforcing compliance through rigid system constraints, this approach supports prescribers through professional dialogue, clinical reasoning, and persuasive communication—mechanisms that may be particularly relevant in promoting acceptance and gradual cultural change in prescribing practices.

We have added a discussion to reflect these considerations and to highlight the need for future integration of structural interventions, as system capacity evolves (Discussion, page 20, lines 385-392).

We appreciate the reviewer’s valuable perspective on the long-term sustainability of such interventions.

REVIEWER 2 COMMENTS

Reviewer 2 Comment 1. Please consider consistently using SAP as an abbreviation in the manuscript. As surgical antibiotic prophylaxis has already been described as SAP in the abstract (Line 19), I would recommend using the abbreviation instead of the full term on Line 34 (..."improved compliance with surgical antibiotic prophylaxis..."), Line 81 and Line 118.

Response: Thank you for pointing out these abbreviation issues.

We have carefully reviewed the manuscript and used SAP as an abbreviation throughout the manuscript (line 37, 85 and 128)

Reviewer 2 Comment 2. Introduction: Line 47 - change appropriateness to appropriate for clarity

Response: Thank you for pointing out these typographical and formatting issues.

We have carefully reviewed the manuscript and corrected all typographical errors and inconsistencies throughout the text

Reviewer 2 Comment 3. On Line 82, there is a reference to the Vietnam Ministry of Health guidelines. I found accessing the guidelines difficult. Would it be possible to briefly mention some of the key points in the guideline (e.g. common antibiotic choice or recommended duration of use) in the manuscript for readers to use a reference when reviewing results of the study?

Response:

We thank the reviewer for this helpful suggestion.

We agree that providing additional context on the Vietnam Ministry of Health guidelines would improve the interpretability of our findings for international readers. In response, we have added a brief summary of key recommendations (including common antibiotic choices and recommended duration of prophylaxis) in the main text (page 04, lines 86–90).

We appreciate the reviewer’s suggestion, which has helped enhance the clarity and accessibility of our manuscript.

Reviewer 2 Comment 4. Line 213: should "phas" be phases?

Response: Thank you for pointing out these typographical and formatting issues.

We have carefully reviewed the manuscript and corrected all typographical errors and inconsistencies throughout the text.

Reviewer 2 Comment 5. Line 280: Change "perspective" to prospective

Response: Thank you for pointing out these typographical and formatting issues.

We have carefully reviewed the manuscript and corrected all typographical errors and inconsistencies throughout the text.

Attachment Submitted filename: Response to editor and reviewers.docx

# Editor report: Decision Letter 1

**DOI:** 10.1371/journal.pone.0351068.r003

**Authors:** Tze Shien Lo, Tze Shien Lo

21 May 2026

Optimizing Surgical Antimicrobial Prophylaxis through Clinical Pharmacist-Led

Audit and Feedback: Evidence from a Vietnamese Tertiary Hospital

PONE-D-25-68518R1

Dear Dr. Son Tu Nguyen

We’re pleased to inform you that your manuscript has been judged scientifically suitable for publication and will be formally accepted for publication once it meets all outstanding technical requirements.

Within one week, you’ll receive an e-mail detailing the required amendments. When these have been addressed, you’ll receive a formal acceptance letter and your manuscript will be scheduled for publication.

An invoice will be generated when your article is formally accepted. Please note, if your institution has a publishing partnership with PLOS and your article meets the relevant criteria, all or part of your publication costs will be covered. Please make sure your user information is up-to-date by logging into Editorial Manager at Editorial Manager® and clicking the ‘Update My Information' link at the top of the page. For questions related to billing, please contact billing support.

If your institution or institutions have a press office, please notify them about your upcoming paper to help maximize its impact. If they’ll be preparing press materials, please inform our press team as soon as possible -- no later than 48 hours after receiving the formal acceptance. Your manuscript will remain under strict press embargo until 2 pm Eastern Time on the date of publication. For more information, please contact onepress@plos.org.

Kind regards,

Tze Shien Lo, MD

Academic Editor

PLOS One

Additional Editor Comments (optional):

Reviewers' comments:

# Editor report: Acceptance letter

**DOI:** 10.1371/journal.pone.0351068.r004

**Authors:** Tze Shien Lo, Tze Shien Lo

PONE-D-25-68518R1

PLOS One

Dear Dr. Nguyen,

I'm pleased to inform you that your manuscript has been deemed suitable for publication in PLOS One. Congratulations! Your manuscript is now being handed over to our production team.

At this stage, our production department will prepare your paper for publication. This includes ensuring the following:

* All references, tables, and figures are properly cited

* All relevant supporting information is included in the manuscript submission,

* There are no issues that prevent the paper from being properly typeset

You will receive further instructions from the production team, including instructions on how to review your proof when it is ready. Please keep in mind that we are working through a large volume of accepted articles, so please give us a few days to review your paper and let you know the next and final steps.

Lastly, if your institution or institutions have a press office, please let them know about your upcoming paper now to help maximize its impact. If they'll be preparing press materials, please inform our press team within the next 48 hours. Your manuscript will remain under strict press embargo until 2 pm Eastern Time on the date of publication. For more information, please contact onepress@plos.org.

You will receive an invoice from PLOS for your publication fee after your manuscript has reached the completed accept phase. If you receive an email requesting payment before acceptance or for any other service, this may be a phishing scheme. Learn how to identify phishing emails and protect your accounts at https://explore.plos.org/phishing.

If we can help with anything else, please email us at customercare@plos.org.

Thank you for submitting your work to PLOS ONE and supporting open access.

Kind regards,

PLOS ONE Editorial Office Staff

on behalf of

Dr. Tze Shien Lo

Academic Editor

PLOS One
